# Variation in Practice Patterns and Reimbursements Between Female and Male Urologists for Medicare Beneficiaries

**DOI:** 10.1001/jamanetworkopen.2019.8956

**Published:** 2019-08-09

**Authors:** Catherine S. Nam, Akanksha Mehta, Jessica Hammett, Frances Y. Kim, Christopher P. Filson

**Affiliations:** 1 Department of Urology, Emory University School of Medicine, Atlanta, Georgia; 2 Winship Cancer Institute, Emory Healthcare, Atlanta, Georgia; 3 Department of Urology, Atlanta Veterans Affairs Medical Center, Decatur, Georgia

## Abstract

**Importance:**

Previous assessments of practice patterns and reimbursements for female urologists relied on surveys or board certification logs. A current evaluation of the geographic distribution and practice patterns by female urologists would reveal contemporary patterns of access for Medicare beneficiaries.

**Objective:**

To characterize the variation in practice patterns and reimbursements by urologist sex and the regional deficiencies in care provided by female urologists.

**Design, Setting, and Participants:**

This population-based cohort study used the publicly available Centers for Medicare & Medicaid Services Provider Payment database to evaluate payments for US urologists. The cohort (n = 8665) included urologists who provided and were paid for 11 or more services to Medicare beneficiaries in 2016. Data collection and analysis were performed from October 3, 2018, through June 19, 2019.

**Main Outcomes and Measures:**

Proportion of female-specific services, payments per beneficiary, and payments per work relative value unit (wRVU) by urologist sex were assessed. Density of female urologists across hospital markets was also identified.

**Results:**

Among the 8665 urologists who received payments in 2016, 7944 (91.7%) were men and 721 (8.3%) were women. Female urologists, compared with male urologists, saw a lower proportion of patients with cancer (mean [SD], 16.3% [9.2%] vs 22.7% [8.8%]; *P* < .001) and a greater proportion of female Medicare beneficiaries (mean [SD], 52.8% [23.2%] vs 24.4% [10.3%]; *P* < .001). Female urologists generated a greater proportion of wRVU from urodynamics (median [IQR], 2.88% [1.26%-4.84%] vs 1.07% [0.31%-2.26%]; *P* < .001) and gynecological operations (median [IQR], 0.68% [0.45%-1.07%] vs 0.41% [0.20%-0.81%]; *P* < .001) than male urologists. In addition, female urologists, compared with their male counterparts, received lower median payments per beneficiary seen ($70.12 [interquartile range (IQR), $60.00-$84.81] vs $72.37 [IQR, $59.63-$89.29]; *P* = .03) and lower payments per wRVU ($58.25 [IQR, $48.39-65.26] vs $60.04 [IQR, $51.93-$67.88]; *P* < .001). One-third (103 [33.7%]) of 306 hospital referral regions had 0 female urologists, and 80 (26.1%) had only 1 female urologist.

**Conclusions and Relevance:**

Female urologists were more likely to provide care for female Medicare beneficiaries, to receive lower payments per wRVU generated and beneficiaries seen, and to be difficult to access in certain geographic areas; these findings have policy-related implications and highlight the regional deficiencies in urological care and reimbursement discrepancies according to urologist sex.

## Key Points

**Question:**

Do practice patterns, reimbursements, and geographic distribution of urologists who treat Medicare beneficiaries differ by urologist sex?

**Findings:**

In this population-based cohort study of 8665 US urologists who received Medicare payments in 2016, statistically significant differences in practice patterns and payments were found for female urologists, including considerable geographic gaps in access to these physicians (ie, one-third of hospital markets had 0 female urologists for Medicare beneficiaries).

**Meaning:**

Female urologists appeared to provide unique care for more female Medicare beneficiaries and to perform more female-specific care, but a wide variation in geographic concentration of urologists based on urologist sex was found.

## Teaser

This cohort study uses the US Centers for Medicare & Medicaid Services Provider Payments database to identify whether sex-based discrepancies exist in clinical practice, reimbursement payments, and geographic distributions in the urological specialty.

## Introduction

The American Urological Association estimates that women compose up 8.5% of practicing urologists in the United States.^1^ With an 11-fold increase among active physicians, urology has seen the greatest growth in female representation compared with all other specialties.^2^ This growth has advantages for patients. For instance, patient surveys have highlighted that patients with urinary incontinence prefer to have sex-concordant urologists.^3^ Cross-sectional studies have also found lower mortality and readmission rates for Medicare beneficiaries treated by female physicians.^4^ In addition, more female urologists are pursuing academic careers and fellowship training, and not just in female pelvic medicine and reconstructive surgery.^5^ Increasing representation of women in urology may also generate tension in a previously male-dominated field. Past surveys revealed that one-quarter of female urologists described being pigeonholed into certain practice patterns.^6^ Female physicians have also reported feeling the pressure to hold prolonged office visits for patients with complex psychosocial issues and other difficult conditions.^7^

Current reports of practice patterns of female urologists rely on survey data with varying response rates.^1,8^ These surveys are sensitive to nonresponder bias, undersampling rural urologists and those without academic affiliation. To our knowledge, studies of broad practice patterns and reimbursement for female urologists have not been performed to date. Furthermore, an up-to-date geographic assessment of the distribution of female urologists in the United States has not been released.

In this study, we used publicly available payment data from the Centers for Medicare & Medicaid Services (CMS) to characterize variation in practice patterns and reimbursement of urologists on the basis of urologist sex. We aim for this study to have policy-related implications, highlighting regional deficiencies in care provided by female urologists and sex-based discrepancies in reimbursement. We hypothesized that female urologists delivered more women-specific urological care (eg, stress urinary incontinence) compared with their male counterparts and that density of female urologists would be lower in rural areas of the United States.

## Methods

### Data Set and Cohort

This analysis used the CMS Provider Payments database, which includes aggregated physician-level data for care delivered to fee-for-service Medicare beneficiaries. Emory University School of Medicine deemed this study exempt from institutional review board oversight because of its use of publicly available deidentified data. Informed consent was also waived by Emory University School of Medicine for this reason. This study followed the Strengthening the Reporting of Observational Studies in Epidemiology (STROBE) reporting guidelines.

To protect patient privacy, we reported payments only if the physician performed a service more than 10 times in a year in a particular practice setting (either facility or office). Service-specific data included number of services, number of unique beneficiaries, mean submitted charge, and mean payment based on Healthcare Common Procedure Coding System (HCPCS) code. Physician-specific data included urologist name, National Provider Identifier, address of primary practice (including zip code), academic degree, specialty, and sex. We linked HCPCS codes to pertinent work relative value units (wRVU) using crosswalk files available on the CMS website. We also linked each urologist to aggregated patient-specific data (eg, proportion of patients with cancer, proportion of female patients) using available files on the CMS website.

The analytic cohort was a convenience sample. It comprised urologists who received payments for fee-for-service care from CMS in 2016.

### Geographic Distribution of Female Urologists

The geographic unit of analysis was the hospital referral region (HRR), which was based on the primary practice zip code for each urologist. The number of female urologists per HRR was identified and the number of female urologists per 50 000 female fee-for-services Medicare beneficiaries in 2016 was calculated using data from the CMS website. We also identified the top 10 HRRs with the greatest density of female urologists as well as the top 5 most populous HRRs with the largest number of female Medicare beneficiaries but without a female urologist in 2016.

### Exposures and Outcomes of Interest for Payment Analysis

We assessed payments, services, and wRVU according to urologist sex. We categorized certain services as urodynamics (*Current Procedural Terminology* [*CPT*] 51725-51792), gynecological operations (*CPT* 56405-58999), and female-specific lower urinary tract symptoms–associated procedures (eTable 1 in the Supplement). Services were deemed either office based or facility based. This data set captured nonphysician mediated services billed under level II HCPCS codes, such as leuprolide acetate injection (*CPT* J9217) or prostate-specific antigen testing (HCPCS code G0103). Evaluation and management services included all care with *CPT* codes 99201-99499. For each urologist sex, we identified the top 20 procedures by total aggregated payments.

### Statistical Analysis

We generated descriptive statistics about characteristics of the urologists in this analytic cohort, and we used appropriate χ^2^ testing to compare differences by urologist sex. We evaluated associations between outcomes, urologist sex, and other factors with bivariate testing. Categorical variables were assessed with Pearson χ^2^ tests. Continuous variables were evaluated with 2-sided tests using either Wilcoxon rank sum (for median values) or 2-tailed, unpaired *t* (for mean values) tests. We considered any *P* < .05 as statistically significant. Statistical testing was carried out with Stata SE, version 14.2 (StataCorp LLC). Data analysis was performed from October 3, 2018, through June 19, 2019.

## Results

We identified 8665 urologists who received Medicare payments in 2016. Of those urologists, 7944 (91.7%) were men and 721 (8.3%) were women (eTable 2 in the Supplement). Most male (7543 [95.0%]) and female (648 [89.9%]) urologists had an MD (doctor of medicine) degree. The largest proportions of female urologists were in the Pacific (12.0%) and New England (11.1%) regions.

The patterns of care for Medicare beneficiaries differed significantly between male and female urologists (Table 1). In general, male urologists compared with their female counterparts saw more Medicare beneficiaries (median [IQR], 562 [331-842] vs 380 [205-577]; *P* < .001) and provided more services (median [IQR], 3665 [1358-8743] vs 2036 [750-4865]; *P* < .001). Male urologists also received a greater amount of per-physician payments in 2016 compared with female urologists (median [IQR], $168 255 [$90 772-$273 060] vs $104 692 [$54 350-$174 792]; *P* < .001). Female urologists, compared with male urologists, saw a lower proportion of patients with cancer (mean [SD], 16.3% [9.2%] vs 22.7% [8.8%]; *P* < .001), had a greater proportion of female Medicare beneficiaries (mean [SD], 52.8% [23.2%] vs 24.4% [10.3%]; *P* < .001) and dual-eligible patients with Medicaid coverage (mean [SD], 20.0% [14.2%] vs 17.3% [14.7%]; *P* < .001), and saw a lower proportion of patients of non-Hispanic white race/ethnicity (mean [SD], 72.6% [28.4%] vs 75.7% [25.1%]; *P* = .002).

**Table 1.** Patterns of Care for Male and Female Urologists Caring for Medicare Beneficiaries

Covariates	Male Urologists (n = 7944)	Female Urologists (n = 721)	*P* Value
2016 Overall productivity, median (IQR)			
Total beneficiaries, No.	562 (331-842)	380 (205-577)	<.001
Total services, No.	3655 (1358-8743)	2036 (750-4865)	<.001
Total payments, US$	168 255 (90 772-273 060)	104 692 (54 350-174 792)	<.001
Total wRVU	3858 (2057-5960)	2573 (1179-4185)	<.001
Patient characteristics, mean (SD), %			
With cancer	22.7 (8.8)	16.3 (9.2)	<.001
Medicaid coverage	17.3 (14.7)	20.0 (14.2)	<.001
Non-Hispanic white race/ethnicity	75.7 (25.1)	72.6 (28.4)	.002
Female	24.4 (10.3)	52.8 (23.2)	.002

Abbreviations: IQR, interquartile range; wRVU, work relative value unit.

Female urologists were more likely to deliver female-specific care, compared with their male counterparts (Table 2). A greater proportion of female than male urologists performed urodynamics (56.0% vs 49.8%; *P* = .001), gynecological operations (17.3% vs 2.7%; *P* < .001), and female incontinence surgical procedures (29.1% vs 16.1%; *P* < .001). Among those who provided the services, female urologists, compared with their male counterparts, had a greater proportion of services associated with urodynamics (median [IQR], 2.99% [1.63%-5.67%] vs 1.89% [0.89%-3.87%]; *P* < .001) and gynecological operations (median [IQR], 0.68% [0.45%-1.07%] vs 0.41% [0.20%-0.81%]; *P* < .001). Similarly, female urologists had a greater proportion of their overall wRVUs associated with urodynamics (median [IQR], 2.88% [1.26%-4.84%] vs 1.07% [0.31%-2.26%]; *P* < .001). Among those performing gynecological operations, no difference was observed in proportion of wRVUs earned between male and female urologists (median [IQR], 2.54% [0.97%-5.00%] vs 2.55% [0.67%-6.27%]; *P* = .86). Among those who provided female-specific incontinence surgical procedures, female urologists had a larger proportion of services (median [IQR], 0.69% [0.39%-1.30%] vs 0.46% [0.25%-0.82%]; *P* < .001) and wRVUs (median [IQR], 2.34% [1.33%-4.35%] vs 1.10% [0.59%-2.36%]; *P* < .001) associated with that service type, compared with male urologists.

**Table 2.** Female-Specific Services and Sex-Based wRVU for Medicare Beneficiaries

Service Type	Male Urologists (n = 7944)		Female Urologists (n = 721)		*P* Value
	No. (%)	Median (IQR), %	No. (%)	Median (IQR), %	
Proportion of services as female-specific among urologists					
Urodynamics	3960 (49.8)	1.89 (0.89-3.87)	404 (56.0)	2.99 (1.63-5.67)	<.001
Gynecological operations	215 (2.7)	0.41 (0.20-0.81)	125 (17.3)	0.68 (0.45-1.07)	<.001
Female urinary incontinence surgical procedures	1277 (16.1)	0.46 (0.25-0.82)	210 (29.1)	0.69 (0.39-1.30)	<.001
Proportion of wRVU as female-specific among urologists					
Urodynamics	3960 (49.8)	1.07 (0.31-2.26)	404 (56.0)	2.88 (1.26-4.84)	<.001
Gynecological operations	215 (2.7)	2.54 (0.97-5.00)	125 (17.3)	2.55 (0.67-6.27)	.86
Female urinary incontinence surgical procedures	1213 (15.3)	1.10 (0.59-2.36)	209 (28.9)	2.34 (1.33-4.35)	<.001

Abbreviations: IQR, interquartile range; wRVU, work relative value unit.

**Figure 1.**
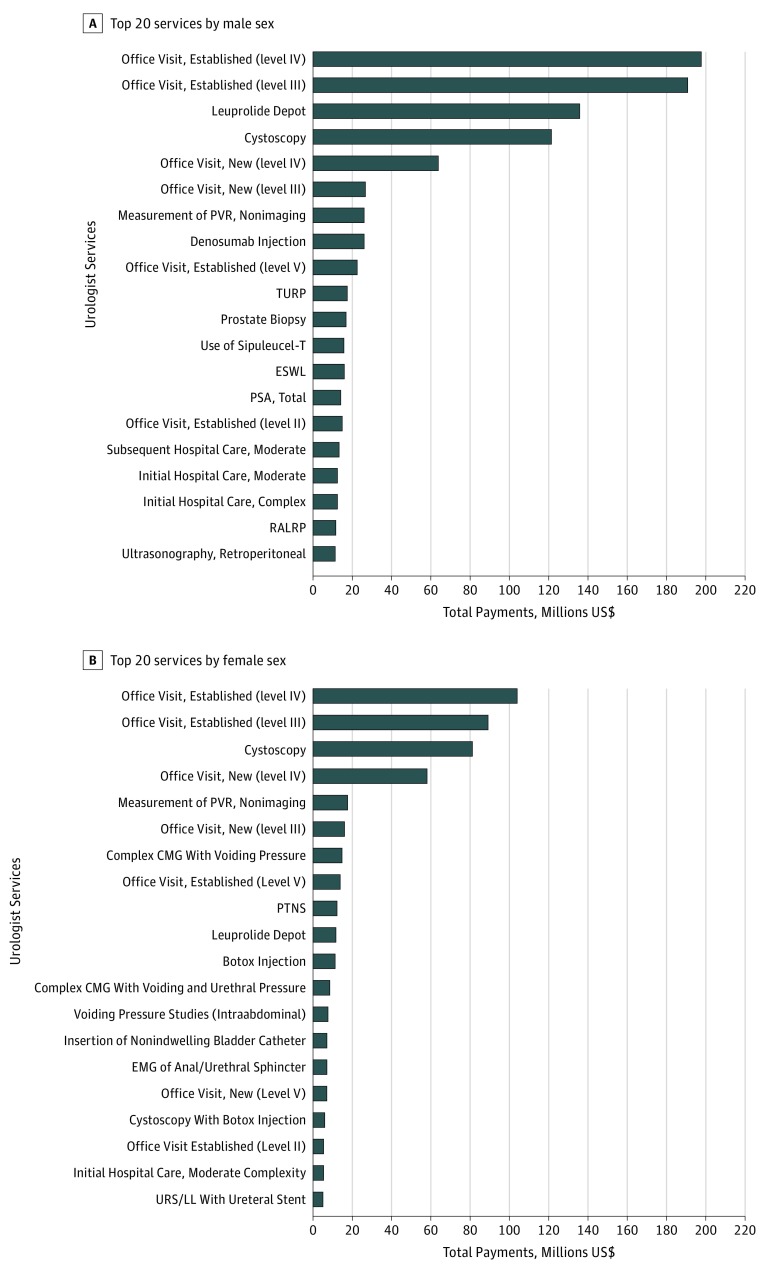
Top 20 Urologist Services by Total Medicare Payments and by Urologist Sex Evaluation and management services were the highest-paying type of services for both male (A) and female (B) urologists (57% vs 60%). The proportion of payments for urodynamics/lower urinary tract symptoms was higher in female compared with male urologists (20% vs 5%). In contrast, the proportion of total payments for oncology was statistically significantly lower for female compared with male urologists (3% vs 23%). CMG indicates cystometrography; EMG, electromyogram; ESWL, extracorporeal shock wave lithotripsy; PSA, prostate-specific antigen; PTNS, percutaneous tibial nerve stimulation; PVR, postvoid residual; RALRP, robotic-assisted laparoscopic radical prostatectomy; TURP, transurethral resection of the prostate; and URS/LL, ureteroscopy/laser lithotripsy.

Figure 1 shows the top 20 services in total payments according to urologist sex. Evaluation and management services were most common for both male and female urologists ($508.4 million [57% of top 20] vs $31.4 million [60% of top 20]). The proportion of payments for urodynamics or lower urinary tract symptoms–associated services was higher in female than male urologists (18% vs 5%). In contrast, the proportion of total payments for oncology-related services was statistically significantly lower for female compared with male urologists (3% vs 23%), primarily owing to substantial use of leuprolide ($124.3 million) and sipuleucel-T ($15.0 million) by male urologists.

In general, female urologists provided a lower proportion of services in the office, compared with male urologists (mean [SD], 77.3% [35.1%] vs 83.1% [27.6%]; *P* < .001). Median (IQR) payments per beneficiary in facility settings were lower for female than for male urologists ($100.35 [$75.29-$148.80] vs $127.55 [$89.60-$188.30]; *P* < .001). Female urologists were less likely to provide services associated with nonphysician-mediated level II HCPCS codes for drugs such as leuprolide or sipuleucel-T injections (mean [SD, 0.5% [1.0%] vs 0.8% [1.8%]; *P* < .001). Accordingly, female urologists had a smaller proportion of payments from level II HCPCS codes (1.8% vs 6.2%; *P* < .001) but received a larger proportion of payments associated with evaluation and management services (28.9% vs 27.1%; *P* < .001), compared with male urologists. Partially owing to the differences in non-wRVU-generating drug administration, female urologists received lower per-wRVU Medicare payments compared with male urologists ($58.25 [interquartile range, (IQR), $48.39-65.26] vs $60.04 [IQR, $51.93-$67.88]; *P* < .001). In addition, female urologists, compared with their male counterparts, received lower payments per beneficiary seen ($70.12 [IQR, $60.00-$84.81] vs $72.37 [IQR, $59.63-$89.29]; *P* = .03) .

**Figure 2.**
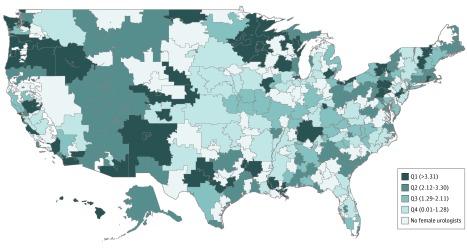
Number of Female Urologists per 50 000 Female Medicare Beneficiaries in the United States The geographic unit of analysis was the hospital referral region (HRR), which is based on the zip code of the primary practice address of each urologist. Data from the Centers for Medicare & Medicaid Services Public Use File (https://www.cms.gov/Research-Statistics-Data-and-Systems/Statistics-Trends-and-Reports/Medicare-Geographic-Variation/GV_PUF.html) were used to find the number of female urologists per HRR and to calculate the number of female urologists per 50 000 female fee-for-service Medicare beneficiaries in 2016. The geographic distribution is color coded by quartile (Q) of urologist density.^9^

The geographic distribution of female urologists per 50 000 female fee-for-service Medicare beneficiaries is shown in Figure 2.^9^ One-third (103 [33.7%]) of 306 HRRs had 0 female urologists, and 80 (26.1%) had only 1 female urologist. The 3 HRRs with the greatest density of female urologists were St Cloud, Minnesota (23.5 female urologists per 50 000 female beneficiaries); Rochester, Minnesota (12.9 female urologists per 50 000 female beneficiaries); and New Orleans, Louisiana (8.0 female urologists per 50 000 female beneficiaries) (eTable 3 in the Supplement). Richmond, Virginia (127 006 female beneficiaries), and Columbia, South Carolina (92 785 female beneficiaries), were the 2 most populous HRRs without a female urologist serving female Medicare beneficiaries (eTable 4 in the Supplement).

## Discussion

This examination of practice and payment patterns among male and female urologists has 3 key findings. First, the patient populations and services associated with female urologists were statistically significantly different from those associated with male urologists. Female urologists, compared with their male counterparts, were more likely to see women, people of color, and Medicaid patients and had a greater share of their practice associated with urodynamics and gynecological operations. Second, considerable geographic gaps in access to female urologists were observed, in which one-third of hospital markets did not have a female urologist to serve Medicare beneficiaries. Third, female urologists received lower total payments per beneficiary and per wRVU compared with male urologists.

When evaluating for patient preference of physicians on the basis of sex, patients tended to have no such preference for instrumental health professionals such as surgeons or anesthesiologists. However, they may have a preference for sex-concordant physicians for more intimate and psychosocial health problems, such as gynecologists or psychiatrists.^10^ Women’s preference for sex-concordant physicians has been found in obstetrics and gynecology literature across diverse patient populations.^11,12,13^ Sex-concordant physician preference for urological conditions has been demonstrated among patients with urinary incontinence.^3^

These preferences may lead to unique practice patterns for female urologists. Female urologists have been shown to perform a greater proportion of female-specific procedures, according to operative logs submitted for board certification. Even though 9.2% of urologists who logged at least 1 case were women, they logged a disproportionate 22.6% of female urological procedures.^14^ This discrepancy could be explained by multiple factors, including referral patterns, patient preferences, and increasing representation by women in the female and pelvic reconstruction subspecialty. The present study affirms the findings in these previous studies by using Medicare payments to urologists, indicating that female urologists performed for Medicare beneficiaries a greater proportion of services that are female specific (eg, urodynamics, gynecological operations, and female urinary incontinence–associated surgical procedures). Although more newly trained female urologists are subspecializing across a variety of disciplines,^15^ the study findings may not entirely be associated with female urologists gaining fellowship training in female pelvic medicine and reconstructive surgery. Survey data suggest that even non–fellowship-trained female general urologists care for a higher proportion of female patients.^16^

Given that women may prefer female urologists, and female urologists may provide greater access to female-centric urological services, equal geographic distribution would be critical to ensure equitable access. Without even taking physician sex into account, deficits in urological care in parts of the United States are well established. Odisho et al^17^ found that 63% of counties in the United States lack a urologist, particularly in nonmetropolitan and rural counties. Furthermore, urologists who practice in more geographically isolated areas with less dense populations have unique practice patterns. More isolated urologists tend to be older, solo practitioners, less specialized, and less likely to perform major urological services.^18^ For female urologists specifically, Saltzman et al^5^ found that the ratio of practicing female urologists in urban to rural setting was 16.5:1, with less than 10% of female urologists working in rural areas. We did not specifically compare rural with urban areas in this analysis, but the map we created showed wide areas of rural Georgia, Nevada, Texas, North and South Dakota, Illinois, and Wyoming as lacking any female urologists. This variability in the sex-specific geographic distribution of the workforce is not unique to urology. For instance, the American College of Obstetricians and Gynecologists found that approximately 49% of US counties lacked a single obstetrician-gynecologist, although more than 10 million women lived in these underserved rural counties in 2010.^19^

Although women currently compose a minority of the urological workforce, the proportion of female urologists is increasing rapidly. As this disparity narrows and female urologists fill the geographic gaps in access in the United States, they may encounter issues with being pigeonholed into more time-intensive or less-lucrative services.^6,7^ A sex-based wage gap has been well documented in medicine. Among specialists, male physicians in general earn approximately 36% more compared with female urologists.^20,21^ Sex-based representation has become more equitable across all specialties, with a higher number of female medical school graduates, but Seabury et al^22^ found that female physicians experienced no statistically significant improvement in earnings compared with male physicians from 1987 to 2010. Within urology, being a woman is a statistically significant factor in lower compensation, after controlling for work hours, call frequency, age, practice setting and type, fellowship training, and advanced practice practitioners employment; Spencer et al^8^ noted that adjusted salaries for women were $75 321 less than those for men.

These disparities are not unique to urology when compared with other surgical subspecialties. Female otolaryngologists were found to earn approximately 20% less after controlling for differences in hours worked, hours performing surgical procedures, type of practice, and years since completion of training.^23^ Even in obstetrics and gynecology, in which 60% of the practicing physicians are women, Weeks and Wallace^24^ found that sex contributed to lower net annual incomes among office-based obstetrician-gynecologists, with income disparity approaching 17%.

Although the wage gap is multifactorial, a proposed factor has been the different practice and billing patterns between female and male urologists. This study supported this explanation, as male urologists were much more likely to be reimbursed for lucrative, often drug-related level II HCPCS services (such as androgen deprivation therapy), which are not associated with wRVU generation. Furthermore, other aspects of care for female patients may decrease efficiency in clinic (eg, time-consuming pelvic examinations, requests for chaperones).

### Limitations

This study has several limitations. First, the CMS database does not include low-volume services of fewer than 11 cases per year per National Provider Identifier. However, given that most reimbursements are for high-volume evaluation and management services, missing low-volume service data are unlikely to have major implications for the findings. Second, generalizability is limited to Medicare beneficiaries. Female urologists may be providing a greater proportion of care for younger patients covered by employer-based insurance or for patients covered by Medicare Advantage plans. We did not capture female urologists who cared solely for patients without Medicare coverage (eg, pediatric urologists). We assumed that we captured nearly all female urologists who provided care to Medicare beneficiaries, regardless of whether the practitioners were hospital employees or in solo or group practice. We only would have excluded those who performed fewer than 10 episodes of any service for this patient population. However, the CMS database represents a substantial portion of urological care in the United States. Third, we did not have access to other physician characteristics that may be factors in different practice patterns. For instance, female practitioners are likely to be younger, in general, compared with male practitioners; thus, their practices may be less mature and show unique patterns, which may change as they gain experience in the field. Fourth, we could not capture care delivered by advanced practice practitioners in this analysis, which is a valuable contribution to urological care in resource-strapped regions.^25^ Future analyses should examine the prevalence of office-based procedures (eg, urodynamics) billed by male and female advanced practice practitioners in the United States.

Assessing the implications of the observed discrepancies in the patterns of care delivered by male and female urologists who treat Medicare beneficiaries is crucial. In particular, knowing whether these differences are narrowing would be helpful to understanding how the growing number of female urologists is associated with sex-based differences in urological practice patterns. In addition, a number of questions remain. What is the association between physician training and experience and urologist sex? That is, would female patients prefer a female nurse practitioner over a male physician? Finally, are male and female urologists billing for similar services when they see similar patients? These questions are not answerable in this analysis but are important to investigate in the future.

## Conclusions

Although the basis of these findings is complex, we observed that female urologists in the United States provided care for more female Medicare beneficiaries and provided more female-specific care compared with male urologists. In this context, the wide geographic variation in density of female urologists (including large areas without coverage by female urologists) highlights an opportunity for policy mechanisms to improve the geographic distribution of female urologists.

## Notes

Supplement. **eTable 1.** Procedure Codes for Urodynamics, Gynecologic Services, and Female-Specific Services **eTable 2.** Characteristics of Cohort of Providers Based on Gender **eTable 3.** Top Ten Hospital Referral Regions in Terms of Density of Female Urologists **eTable 4.** Five Largest Hospital Referral Regions in Terms of Population of Female Fee-for-Service Medicare Beneficiaries Without a Female Urologist
