# Supplementary material for: Variation in Practice Patterns and Reimbursements Between Female and Male Urologists for Medicare Beneficiaries
Source: JAMA Netw Open. 2019 Aug 9;2(8):e198956. doi: 10.1001/jamanetworkopen.2019.8956 (PMC6692839; doi:10.1001/jamanetworkopen.2019.8956)
Supplement: Supplement. — eTable 1. Procedure Codes for Urodynamics, Gynecologic Services, and Female-Specific Services eTable 2. Characteristics of Cohort of Providers Based on Gender eTable 3. Top Ten Hospital Referral Regions in Terms of Density of Female Urologists eTable 4. Five Largest Hospital Referral Regions in Terms of Population of Female Fee-for-Service Medicare Beneficiaries Without a Female Urologist [file jamanetwopen-2-e198956-s001.pdf]

Supplementary Online Content

Nam CS, Mehta A, Hammett J, Kim FY, Filson CP. Variation in practice patterns and reimbursements between female and male urologists for Medicare beneficiaries. *JAMA Netw Open*. 2019;2(8): e198956. doi:10.1001/jamanetworkopen.2019.8956

eTable 1. Procedure Codes for Urodynamics, Gynecologic Services, and Female-Specific Services

eTable 2. Characteristics of Cohort of Providers Based on Gender

eTable 3. Top Ten Hospital Referral Regions in Terms of Density of Female Urologists

eTable 4. Five Largest Hospital Referral Regions in Terms of Population of Female Fee-for-Service Medicare Beneficiaries Without a Female Urologist

This supplementary material has been provided by the authors to give readers additional information about their work.

©2019 Nam CS et al. *JAMA Network Open*

eTable 1. Procedure Codes for Urodynamics, Gynecologic Services, and Female-Specific Services

HCPCS	Procedure
20926	Martius flap
51715	Endoscopic injection into urethra/bladder neck
51725 – 51792	Urodynamics
51840	Anterior vesicourethropexy; simple
51841	Anterior vesicourethropexy; complicated
51845	Abdomino-vaginal vesical neck suspension
51900	Closure vesicovaginal fistula
51920	Closure vesicouterine fistula
51925	Closure of vesicouterine fistula with hysterectomy
51990	Laparoscopy; urethral suspension for stress incontinence
51992	Laparoscopy; sling operation for stress incontinence
51999	Laparoscopy; unlisted, bladder
52260	Cystoscopy/dilation for interstitial cystitis
52270	Cystourethroscopy with internal urethrotomy, female
52285	Cystourethroscopy for treatment of female urethral syndrome
52287	Cystoscopy with injection for denervation of bladder
53060	Incision and drainage of Skene's gland abscess
53230	Excision urethral diverticulum, female
53240	Marsupialization of urethral diverticulum
53270	Excision/fulguration of Skene's glands
53275	Excision/fulguration of urethral prolapse
53500	Urethrolysis, transvaginal
53660	Dilation of female urethra, initial
53661	Dilation of female urethra, subsequent
53665	Dilation of female urethra, under general anesthesia
53860	Transurethral radiofrequency (RF) microremodeling of female urethra for stress urinary incontinence
56000 – 58999	Gynecologic operations
64561	Percutaneous implantation of neurostimulator electrode, sacral
64566	Percutaneous tibial neurostimulation
64581	Incision for implantation of neurostimulator electrode, sacral
64590	Insertion/replacement of peripheral neurostimulator
64595	Revision/removal of peripheral neurostimulator

©2019 Nam CS et al. *JAMA Network Open*

eTable 2. Characteristics of Cohort of Providers Based on Gender (Total n=8,665)

	Male Urologists (n= 7,944)	Female Urologists (n=721)	p
Credentials			<0.001
MD	7,543 (92)	648 (8)	
DO/MBBS/Other	289 (84)	54 (16)	
Unknown	112 (85)	19 (15)	
Region			<0.001
Pacific	945 (88)	129 (12)	
Mountain	472 (90)	53 (10)	
W North Central	517 (91)	52 (9)	
W South Central	844 (92)	75 (8)	
E North Central	1,191 (92)	106 (8)	
E South Central	481 (92)	41 (8)	
South Atlantic	1,726 (94)	104 (6)	
Middle Atlantic	1,326 (93)	106 (7)	
New England	442 (89)	55 (11)	
Practice Site			<0.001
Facility and Office	6,276 (93)	481 (7)	
Office only	1,118 (89)	144 (11)	
Facility only	550 (85)	96 (15)	

©2019 Nam CS et al. *JAMA Network Open*

eTable 3. Top Ten Hospital Referral Regions in Terms of Density of Female Urologists

HRR	City/State	Female Urologists per 50,000 Female Beneficiaries
254	St Cloud, MN	23.49
253	Rochester, MN	12.89
218	New Orleans, LA	8.01
341	Bend, OR	7.54
304	Rochester, MN	7.13
399	Longview, TX	6.46
439	Seattle, WA	5.95
232	Ann Arbor, MI	5.74
256	St Paul, MN	5.69
342	Eugene, OR	5.42

©2019 Nam CS et al. *JAMA Network Open*

eTable 4. Five Largest Hospital Referral Regions in Terms of Population of Female Fee-for-Service Medicare Beneficiaries Without a Female Urologist

HRR	City/State	# Female Beneficiaries
431	Richmond, VA	127,006
366	Columbia, SC	92,785
285	Morristown, NJ	76,229
172	Springfield, IL	72,232
112	Wilmington, DE	67,279

©2019 Nam CS et al. *JAMA Network Open*
